# Bortezomib restrains M2 polarization and reduces CXCL16-associated CXCR6^+^CD4 T cell chemotaxis in bleomycin-induced pulmonary fibrosis

**DOI:** 10.1186/s10020-024-00836-5

**Published:** 2024-05-24

**Authors:** Ting Zhou, Lan lin, Yawen Zhan, Ziyao Zhang, Ying Jiang, Mi Wu, Dan Xue, Limin Chen, Xiufang Weng, Zhenghui Huang

**Affiliations:** 1 Department of Immunology, School of Basic Medicine, Tongji Medical College, Huazhong University of Science and Technology, Wuhan, 430030 China; 2 Department of Pulmonary and Critical Care Medicine, Fujian Medical University Union Hospital, Fuzhou, 350001 China

**Keywords:** Pulmonary fibrosis, Bleomycin, Bortezomib, CXCR6, CXCL16, Macrophage, CD4 T cell

## Abstract

**Background:**

The development of pulmonary fibrosis involves a cascade of events, in which inflammation mediated by immune cells plays a pivotal role. Chemotherapeutic drugs have been shown to have dual effects on fibrosis, with bleomycin exacerbating pulmonary fibrosis and bortezomib alleviating tissue fibrotic processes. Understanding the intricate interplay between chemotherapeutic drugs, immune responses, and pulmonary fibrosis is likely to serve as the foundation for crafting tailored therapeutic strategies.

**Methods:**

A model of bleomycin-induced pulmonary fibrosis was established, followed by treatment with bortezomib. Tissue samples were collected for analysis of immune cell subsets and functional assessment by flow cytometry and in vitro cell experiments. Additionally, multi-omics analysis was conducted to further elucidate the expression of chemokines and chemokine receptors, as well as the characteristics of cell populations.

**Results:**

Here, we observed that the expression of CXCL16 and CXCR6 was elevated in the lung tissue of a pulmonary fibrosis model. In the context of pulmonary fibrosis or TGF-β1 stimulation in vitro, macrophages exhibited an M2-polarized phenotype and secreted more CXCL16 than those of the control group. Moreover, flow cytometry revealed increased expression levels of CD69 and CXCR6 in pulmonary CD4 T cells during fibrosis progression. The administration of bortezomib alleviated bleomycin-induced pulmonary fibrosis, accompanied by reduced ratio of M2-polarized macrophages and decreased accumulation of CD4 T cells expressing CXCR6.

**Conclusions:**

Our findings provide insights into the key immune players involved in bleomycin-induced pulmonary fibrosis and offer preclinical evidence supporting the repurposing strategy and combination approaches to reduce lung fibrosis.

**Supplementary Information:**

The online version contains supplementary material available at 10.1186/s10020-024-00836-5.

## Background

Pulmonary fibrosis is a complex disease spectrum often associated with various disorders. This condition involves progressive scarring of lung tissue, leading to impaired respiratory function and an unfavorable prognosis (Koudstaal et al. 2023). The onset of drug-induced pulmonary fibrosis has been linked to specific medications, including chemotherapeutic and rheumatologic drugs (McGee et al. 2018). The association between chemotherapeutic drugs and pulmonary fibrosis, particularly within the realm of cancer treatment, is notable. While chemotherapeutic agents are essential for combating tumors, certain drugs, such as bleomycin, are well-known for their propensity to induce pulmonary fibrosis. Bleomycin is often used to treat various cancers, including Hodgkin’s lymphoma and germ cell tumors (Watson et al. 2018; Hecht 2000; Shanbhag and Ambinder 2018), however, its utility is limited by the risk of inducing lung injury and pulmonary fibrosis (Della Latta et al. 2015). The pro-fibrosis side effect of bleomycin has led to its widespread use in inducing pulmonary fibrosis models (Liu et al. 2017). Thus, thorough monitoring and management of pulmonary complications during bleomycin therapy remain imperative. Conversely, bortezomib, a proteasome inhibitor primarily used for managing multiple myeloma, has exhibited anti-fibrotic effects across various tissues, hinting at its potential for preventing dermal, renal, and pulmonary fibrosis (Koca et al. 2012; Zeniya et al. 2017; Mutlu et al. 2012). The intricate relationship between chemotherapeutic drugs and pulmonary fibrosis underscores the need for tailored strategies to mitigate these adverse effects. Identifying the underlying mechanisms of drug-induced pulmonary fibrosis and implementing targeted interventions can optimize cancer treatments according to individual patient profiles, thereby reducing the incidence of pulmonary complications.

While pulmonary fibrosis can be triggered by diverse underlying causes, immune-mediated inflammation has consistently emerged as a central mechanism in its pathogenesis. The intricate interplay between immune cell populations—such as macrophages, T cells and other immune effectors—and alveolar epithelial cells and fibroblasts actively contributes to the development of chronic inflammation within the lung microenvironment. This dynamic interaction involves a complex network of signaling molecules, such as cytokines and chemokines, which orchestrate immune cell recruitment and activation, thereby influencing inflammatory and fibrotic responses at local tissue sites. Despite extensive research, the specific immune cell populations and precise mechanisms responsible for sustaining inflammation in pulmonary fibrosis, including bleomycin-induced pulmonary inflammation, remain elusive. Elucidating the pivotal players in inflammation holds promise for advancing our understanding of pulmonary fibrosis pathogenesis and developing targeted therapies to address the specific challenges posed by bleomycin-induced lung injury.

Chemotherapeutic drugs are commonly employed either in combination or as integral components of a comprehensive treatment regimen to enhance the overall effectiveness of cancer treatment. Given the pro-fibrotic effect of bleomycin and the anti-fibrosis potential of bortezomib, there is a rationale to propose a combined regimen of these two drugs, aiming to attain a heightened anti-cancer effect while simultaneously mitigating proinflammatory lung injury. However, the specific role of bortezomib in immune-mediated inflammation in the context of bleomycin-induced pulmonary fibrosis remains unclear.

In the present study, we investigated CXCL16-CXCR6 axis-associated cellular interplay in bleomycin-induced pulmonary fibrosis. Bortezomib intervention significantly alleviated fibrosis, and reduced M2 polarization and CXCL16 expression of macrophage. Additionally, bortezomib treatment attenuated the chemotaxis of CXCR6^+^CD4 T cells in fibrotic lung tissues. Our findings offer the preclinical evidence for a repurposing strategy of bortezomib and suggest a potential combination approach with bleomycin and bortezomib in specific cases to reduce lung fibrosis.

## Materials and methods

### Animal studies

C57BL/6 J male mice aged 6–8 weeks were purchased from Vital River Laboratories (China) and bred in a specific pathogen-free environment. The light and dark conditions were maintained at 12 h each, and the temperature was kept at about 24℃ with the humidity set at approximately 55% ± 5%. Bleomycin (MCE, China) or isopycnic vehicle was intratracheally administrated to the mice at a single dose of 2.5 U/kg. Beginning on the 9th day after bleomycin challenge, bortezomib (0.25 mg/kg, MCE, China) or isopycnic saline was intraperitoneally injected into the mice every 3 days. Mice were divided into the following groups: vehicle (Veh), bortezomib (BTZ), bleomycin + vehicle (BLM), bleomycin + bortezomib (BLM + BTZ). On the 21st day, the mice were euthanized for tissue collection.

### Isolation and culture of bone marrow-derived macrophages

Isolation of murine bone marrow cells and culture of bone marrow-derived macrophages were conducted as previously described (Toda et al. 2021). Briefly, C57BL/6J male mice were euthanized, and femurs and tibias were separated to flush bone marrow cells in precooled PBS. The isolated cells were incubated in erythrocyte lysis buffer and then washed with PBS. After centrifugation at 200 g for 5 minutes at 4°C, the cells were resuspended in RMPI medium (Gibco, USA) supplemented with 10% fetal bovine serum (Gibco, USA), 1% penicillin-streptomycin, and 50 ng/ml recombinant macrophage colony-stimulating factor (M-CSF, PeproTech, USA) and incubated in a cell incubator with 5% CO_2_ at 37℃. After 7 days, mouse bone marrow-derived macrophages were harvested and stimulated with murine TGF-β1 (1 ng/ml or 5 ng/ml, PeproTech, USA) and bortezomib (5 nM).

### HE staining, Masson’s Trichrome staining and IHC staining

The left lung tissues of the mice were collected, fixed in 4% paraformaldehyde, embedded, and sectioned to prepare paraffin sections. The sections were stained with hematoxylin–eosin or performed Masson’s trichrome staining according to instructions.

The procedure used for IHC staining has been described previously (Zhang et al. 2017). Deparaffinized sections were rehydrated and antigen retrieval was achieved by immersion in sodium citrate buffer. After inhibiting endogenous peroxidase activity and blocking non-specific sites with 3% BSA, the slides were incubated with an anti-CXCL16 primary antibody (Absin, China) at 4 °C overnight. IHC staining was performed with an HRP-conjugated secondary antibody followed by DAB detection. Each section was scanned by Pannoramic MIDI (3D HISTECH).

### Preparation of pulmonary single cell suspension, and flow cytometry

Single-cell suspensions of mouse lung tissue were prepared as previously described (Tsukui et al. 2020). Briefly, lung tissues were perfused with PBS through the right ventricle and then cut into pieces for digestion in Liberase TL (Sigma) and DNase I (Sigma) solution at 37 °C for 30 min. Then the cells were passed through a 70 μm cell strainer, washed, and suspended in precooled PBS with 1% fetal bovine serum. For lymphocyte isolation, density gradient centrifugation was performed using Percoll (Cytiva, USA). Before staining with antibodies, the cells were blocked with an anti-CD16/32 antibody (clone 93, BioLegend, USA). Viability was assessed using the Zombie NIR™ Fixable Viability kit (BioLegend, USA). All of the following fluorescence-conjugated antibodies were purchased from BioLegend and eBioscience (USA): anti-CD45(30-F11), anti-TCR-β (H57-597), anti-CD4 (RM4-5), anti-CD8 (53-6.7), anti-CD69 (H1.2F3), anti-CXCR6 (3A051D1), anti-CD11b (M1/70), anti-Ly6G(1A8), anti-F4/80 (BM8), anti-iNOS (CXNFT), and anti-ARG1 (A1exF5). The iNKT cells were stained with fluorescence-conjugated mCD1d/PBS-57 tetramers provided by the National Institutes of Health (NIH) tetramer facility. Data were obtained from FACSVerse (BD Biosciences) and analyzed with FlowJo software (Tree Star).

### ELISA

The culture supernatants of BMDMs and pulmonary cells from the mice were collected. For stimulation of pulmonary cells CXCL16 secretion, phorbol myristate acetate (PMA, 25 ng/ml, Sigma) and ionomycin (Ion, 500 ng/ml, Sigma) were added for 4 h. The CXCL16 level in the supernatants was detected using a mouse CXCL16 ELISA Kit (MULTI SCIENCES, China) as indicated. The optical density (OD) values at 450 nm were measured on a microplate reader, and the concentrations of the samples were determined based on the standard concentration curve.

### Quantitative real-time PCR

Total cellular RNA was isolated using TRIzol Reagent (Agbio, China) and reverse transcribed to cDNA using the First-strand cDNA Synthesis Kit (Vazyme, China) according to the manufacturer’s instructions. Afterward, real-time PCR was performed using TB Green Premix Ex Taq (Takara, Japan) with a CFX96 Real-Time PCR Detection System (Bio-Rad, USA). The relative expression levels of the target genes were normalized to the expression level of the housekeeping gene β-actin. The sequences of the qPCR primers used were as follows: β-actin forward 5-GGCTGTATTCCCCTCCATCG-3, β-actin reverse 5-CCAGTTGGTAACAATGCCATGT-3, ARG1 forward 5-GAACTGAAAGGAAAGTTCCCA-3, ARG1 reverse 5-AATGTACACGATGTCTTTGGC-3, CXCL16 forward 5-TCGCTGGAAGTTGTTCTTGTGA-3, CXCL16 reverse 5-GACCAGTTCCACACTCTTTGCG-3.

### RNA-seq and scRNA-seq dataset analysis

To investigate the role of the chemotaxis pathway in BLM-induced pulmonary fibrosis, GSE213709 (comprising 5 mice administrated with BLM and 5 mice administrated with vehicle) RNA-seq dataset was obtained from the GEO database (https:/www.ncbi.nlm.nih.gov/). To execute GSEA, the “clusterProfiler” (4.8.2) package was used and an adjusted P value < 0.05 was considered.to indicate statistical significance. GSE141259 (comprising mice administered saline only and exposed to BLM for 21 days) scRNA-seq dataset was obtained to analyze the expression of CXCR6 and CXCL16 in pulmonary cell populations. Analysis was performed using the “Seurat” (4.3.0.1) package and gene expression was visualized according to the Seurat “DotPlot” function.

### Statistical analysis

GraphPad Prism 8.0. (USA) was used for the statistical analysis. Data were analyzed by unpaired two-tailed t-tests and Mann‒Whitney tests for comparisons between two groups. Correlation analyses were performed using Pearson correlation. The data were shown as the mean ± SEM and each experiment was replicated at least three times. Differences between groups were considered to be statistically significant when the P value was lower than 0.05.

## Results

### Upregulation of the CXCR6-CXCL16 axis is observed in BLM-induced pulmonary fibrosis and is associated with elevated TGF-β1 levels

**Fig. 1:**
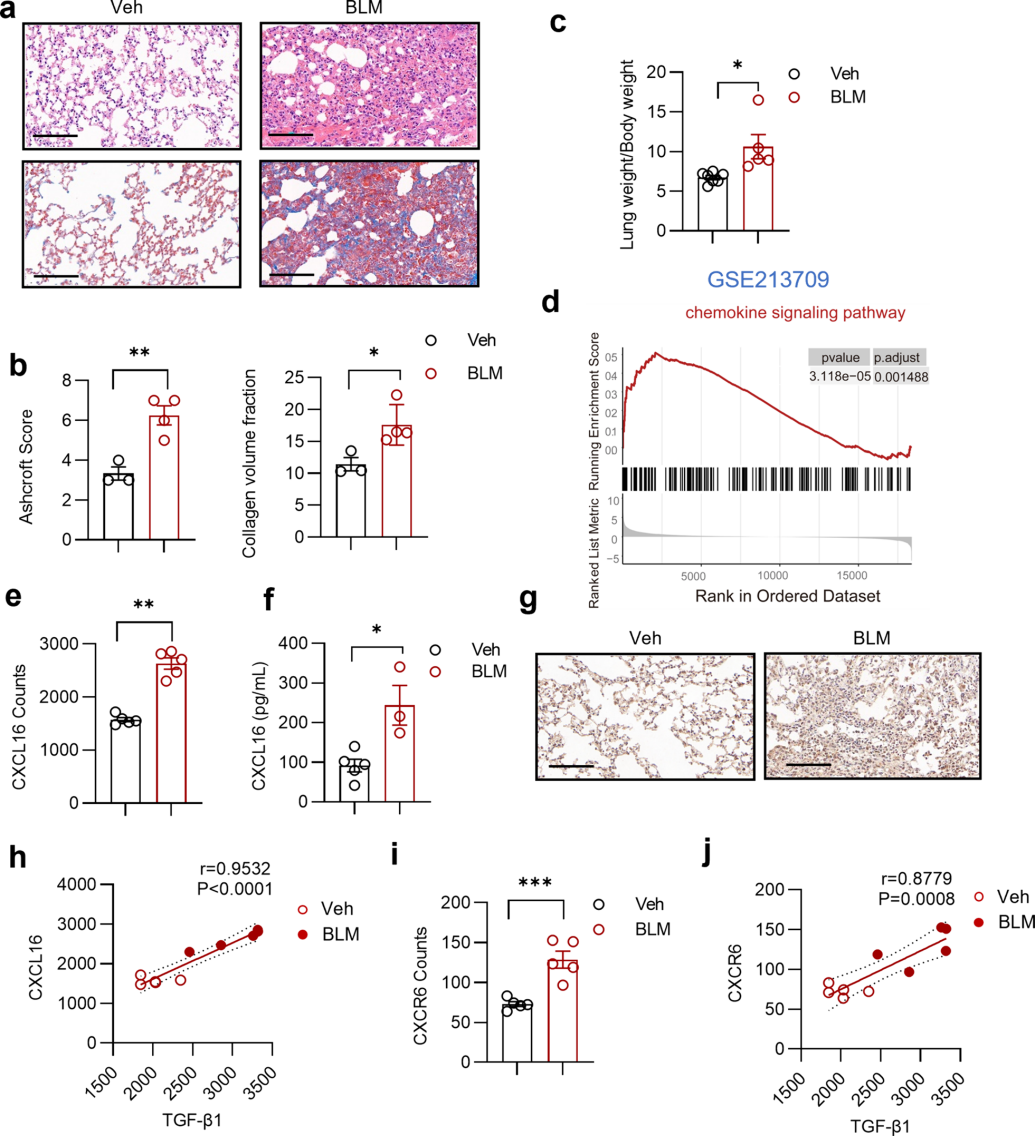
Upregulation of CXCR6-CXCL16 axis in BLM-induced pulmonary fibrosis model. **a** Representative sections of H&E staining and Masson’s Trichrome staining. Scale bar = 100 μm. **b** Summary bar graphs with scatter plots showing Ashcroft score evaluation of H&E staining and collagen volume fraction of Masson’s Trichrome staining. **c** Summary bar graphs with scatter plots showing the lung weight (mg) /body weight (g) index evaluation of indicated groups. **d** The GSEA plot showing chemokine signaling pathway in BLM group versus Veh group (analysis using GSE213709). **e** Summary bar graphs with scatter plots showing CXCL16 mRNA expression in lung tissues of BLM group versus Veh group (analysis using GSE213709). **f** Summary bar graphs with scatter plots showing CXCL16 secretion of PMA-stimulated pulmonary cell supernatant in indicated groups. **g** Representative lung tissue sections of IHC staining of CXCL16. Scale bar = 100 μm. **h** The correlation plot showing the association of CXCL16 and TGF-β1 expression. (analysis using GSE213709) (i) Summary bar graphs with scatter plots showing CXCR6 mRNA expression in lung tissues of BLM group versus Veh group (analysis using GSE213709) **j** The correlation plot showing the association of CXCR6 and TGF-β1 expression. (analysis using GSE213709) *P < 0.05, **P < 0.01, ***P < 0.001. Veh, vehicle, BLM, bleomycin. Data are represented as mean ± SEM

As described by Li et al. (2022), we intratracheally administrated bleomycin to C57BL/6 male mice and sacrificed them on the 21st day. Evaluation of the lung weight/body weight index, HE staining, and Masson’s trichrome staining revealed that revealed evidence of fibrosis progression in the BLM group, characterized by an increased lung weight/body weight index, thickening of alveolar septa, inflammatory cell infiltration, and collagen deposition (Fig. 1a–c). The accumulation or recruitment of immune cells, controlled by interaction between chemokines and chemokine receptors, is a crucial factor in immune cell-mediated inflammation and fibrosis. To decipher the immune milieu in pulmonary fibrosis, we first analyzed the GEO dataset (GSE213709) and observed upregulation of the chemokine signaling pathway (Fig. 1d) along with markedly increased mRNA expression of CXCL16 in the pulmonary fibrosis model (Fig. 1e). Consistent with the transcriptome data, immunohistochemical staining revealed elevated CXCL16 protein expression in fibrotic lung tissue, and the concentrations of CXCL16 in culture supernatants from pulmonary cells were markedly greater in the BLM-treated group than in the saline-treated group (Fig. 1f, g). Moreover, CXCL16 mRNA expression was significantly positively related to TGFB1 mRNA expression (Fig. 1h). The mRNA expression of CXCR6, the only known receptor for CXCL16, in the lung tissues of the BLM group was significantly greater than that in the lung tissues of the Veh group. Additionally, CXCL16 levels were significantly positively correlated with TGFB1 levels (Fig. 1i, j). Although other chemokine-receptor pairs, such as CCL1, CCL8, and CCR8, garnered our attention, these findings suggest the intriguing possibility that the CXCR6-CXCL16 axis may play a role in specific immune cell recruitment and contribute to the pathogenesis of pulmonary fibrosis.

### Macrophages exhibit enhanced M2 polarized phenotype and elevated CXCL16 expression in BLM-induced pulmonary fibrosis

**Fig. 2:**
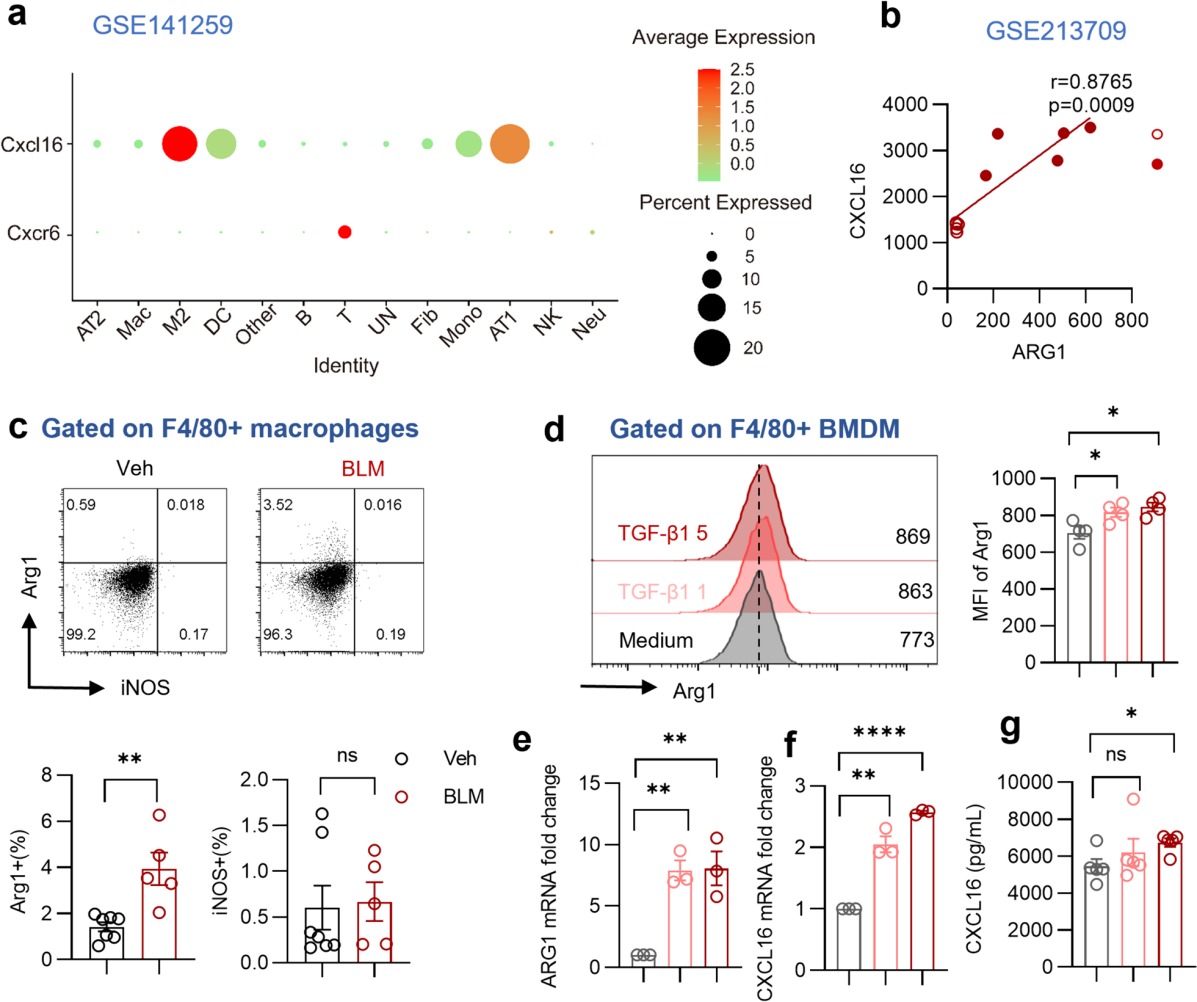
Increased M2-polarized macrophages secret CXCL16 in pulmonary fibrosis. **a** The dot plot of CXCL16 and CXCR6 gene expression in pulmonary cells of scRNAseq dataset (GSE141259, mice administrated with bleomycin or vehicle for 21 days). **b** The correlation plot showing the association of CXCL16 and ARG1 expression. (analysis using GSE213709) **c** Representative plots and summary bar graphs with scatter plots depicting the ratio of iNOS or Arg1 in pulmonary F4/80^+^ macrophages from indicated groups. **d** Representative plots and summary bar graphs with scatter plots showing mean fluorescence intensity of Arg1 in F4/80^+^ BMDMs treated with 0, 1, 5 ng/ml recombinant murine TGF-β1. **e** Summary bar graphs with scatter plots of ARG1 mRNA expression in F4/80^+^ BMDMs treated with 0, 1, 5 ng/ml recombinant murine TGF-β1. (f) Summary bar graphs with scatter plots of CXCL16 mRNA expression in F4/80^+^ BMDMs treated with 0, 1, 5 ng/ml recombinant murine TGF-β1. **g** Summary bar graphs with scatter plots showing CXCL16 secretion of BMDM supernatant in indicated groups. *P < 0.05, **P < 0.01, ****P < 0.0001, ns, no significance. *Veh* vehicle, *BLM* bleomycin, *MFI* mean fluorescence intensity. Data are represented as mean ± SEM

To elucidate the primary cellular sources of CXCR6 and CXCL16, we analyzed gene expression levels in different pulmonary cell populations using a single-cell RNA sequencing dataset (GSE141259, containing mice administrated with bleomycin or vehicle for 21 days). As shown in the dot plot, M2-polarized macrophages expressed the highest CXCL16 level while alveolar epithelial cells only exhibited moderate CXCL16 expression (Fig. 2a). As expected, T cells exhibited high levels of CXCR6 expression, consistent with previous reports (Wein et al. 2019; Korbecki et al. 2021). Consistently, ARG1, a representative marker of M2-polarized macrophage, showed a positive correlation with CXCL16 gene expression (Fig. 2b). Flow cytometry analysis revealed that compared with those in the Veh group, pulmonary F4/80^+^ macrophages of the BLM group expressed more Arg1 but not iNOS, a canonical marker of M1-like macrophages (Fig. 2c). The gating strategy was shown in Supplementary Fig. 1. To simulate the fibrotic environment in vitro, BMDMs were stimulated with recombinant murine TGF-β1 (Liao et al. 2020; Zhang et al. 2016). Flow cytometry and RT-qPCR results confirmed that TGF-β1 treatment enhanced mRNA and protein expression of Arg1 in BMDMs. Elevated CXCL16 mRNA expression was observed in BMDMs stimulated with TGF-β1, and this increase was accompanied by an increase in CXCL16 protein secretion into the culture supernatant of TGF-β1-stimulated BMDMs compared to that of control BMDMs (Fig. 2d–g). Collectively, the above results indicate that in the context of fibrosis, macrophages polarize to the M2 phenotype and exhibit increased levels of CXCL16.

### CD4 T cells accumulate in lung tissue with heightened activation marker and CXCR6 expression in BLM-induced pulmonary fibrosis

**Fig. 3:**
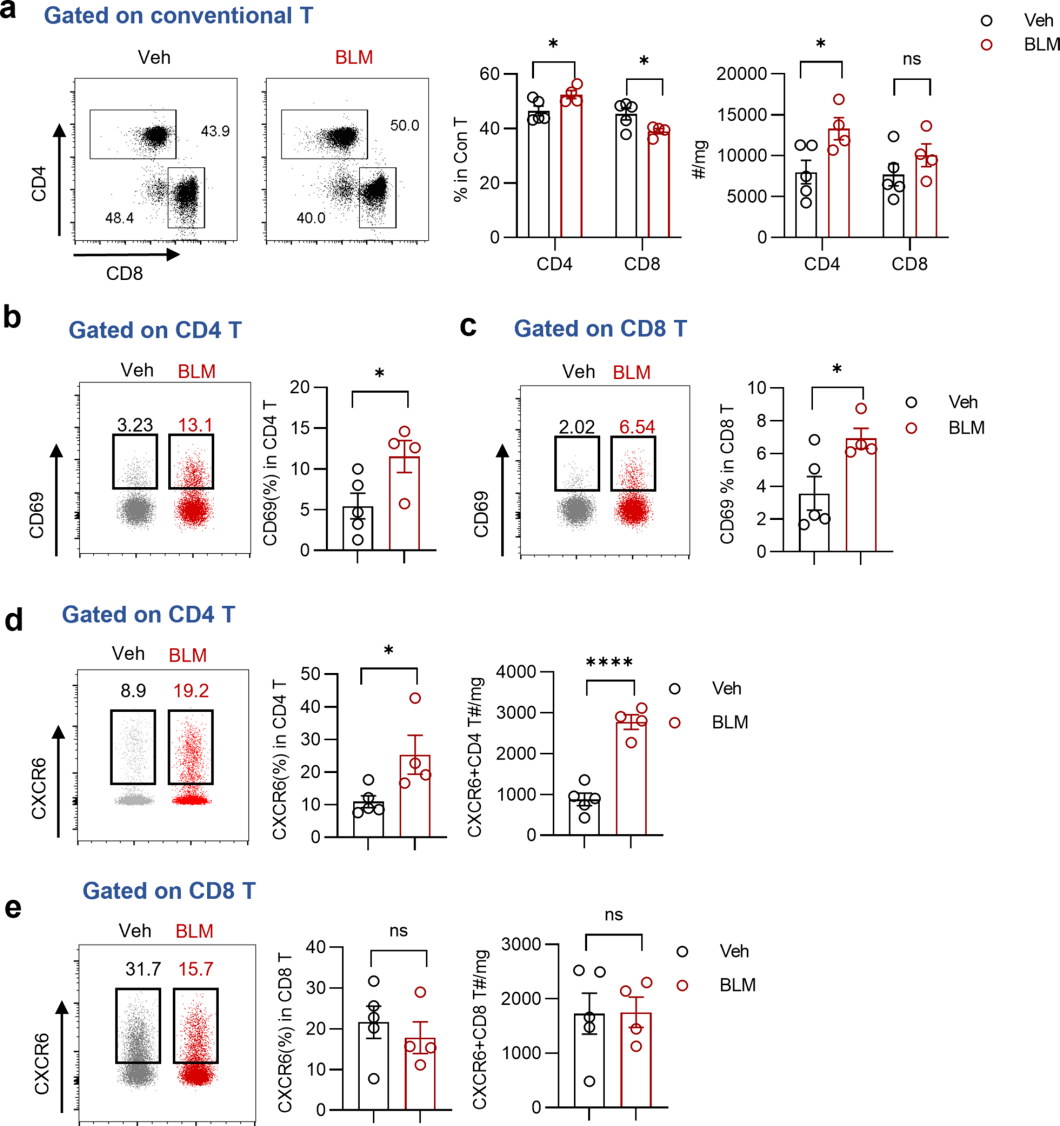
Enhanced accumulation and activation of CD4 T cells in BLM-induced pulmonary fibrosis. **a** Representative plots and summary bar graphs with scatter plots depicting the ratio and number of CD4 T or CD8 T in pulmonary conventional T cells from indicated groups. **b**, **c** Representative plots and summary bar graphs with scatter plots showing CD69 expression of CD4 T cells and CD8 T cells from indicated groups respectively. **d**, **e** Representative plots and summary bar graphs with scatter plots depicting the ratio and number of CXCR6^+^ CD4 T cells and CD8 T cells respectively. *P < 0.05, ****P < 0.0001, ns, no significance. *Veh* vehicle, *BLM* bleomycin. Data are represented as mean ± SEM

In response to CXCL16, CXCR6^+^T cells were speculated to be recruited to fibrotic lung tissues. To further elucidate the changes in the number, activation status, and CXCR6 expression of T cell subpopulations, we next analyzed pulmonary lymphocytes by flow cytometry. The gating strategy was shown in Supplementary Fig. 1. After excluding invariant natural killer T (iNKT) cells to gate conventional T cells, we observed an elevated cell ratio and number of lung CD4 T cells, but not CD8 T cells in mice with pulmonary fibrosis (Fig. 3a). Furthermore, CD69 expression in both CD4 T cells and CD8 T cells in fibrotic lung tissues was upregulated (Fig. 3b, c), suggesting that most T cells are activated during fibrosis progression. Additionally, we observed a significant increase in the percentage and quantity of CXCR6^+^CD4 T cells in the BLM group compared to those in the Veh group, whereas similar findings were not observed in CD8 T cells (Fig. 3d, e). Taken together, these findings indicate that pulmonary CD4 T cells, but not CD8 T cells, exhibit significant increases in both quantity and ratio, along with heightened activation in the context of fibrosis. Moreover, CXCR6-expressing CD4 T cells are recruited to fibrotic lung tissues.

### Bortezomib reduces CXCL16 expression in lung tissue and relieves bleomycin-induced pulmonary fibrosis

**Fig. 4:**
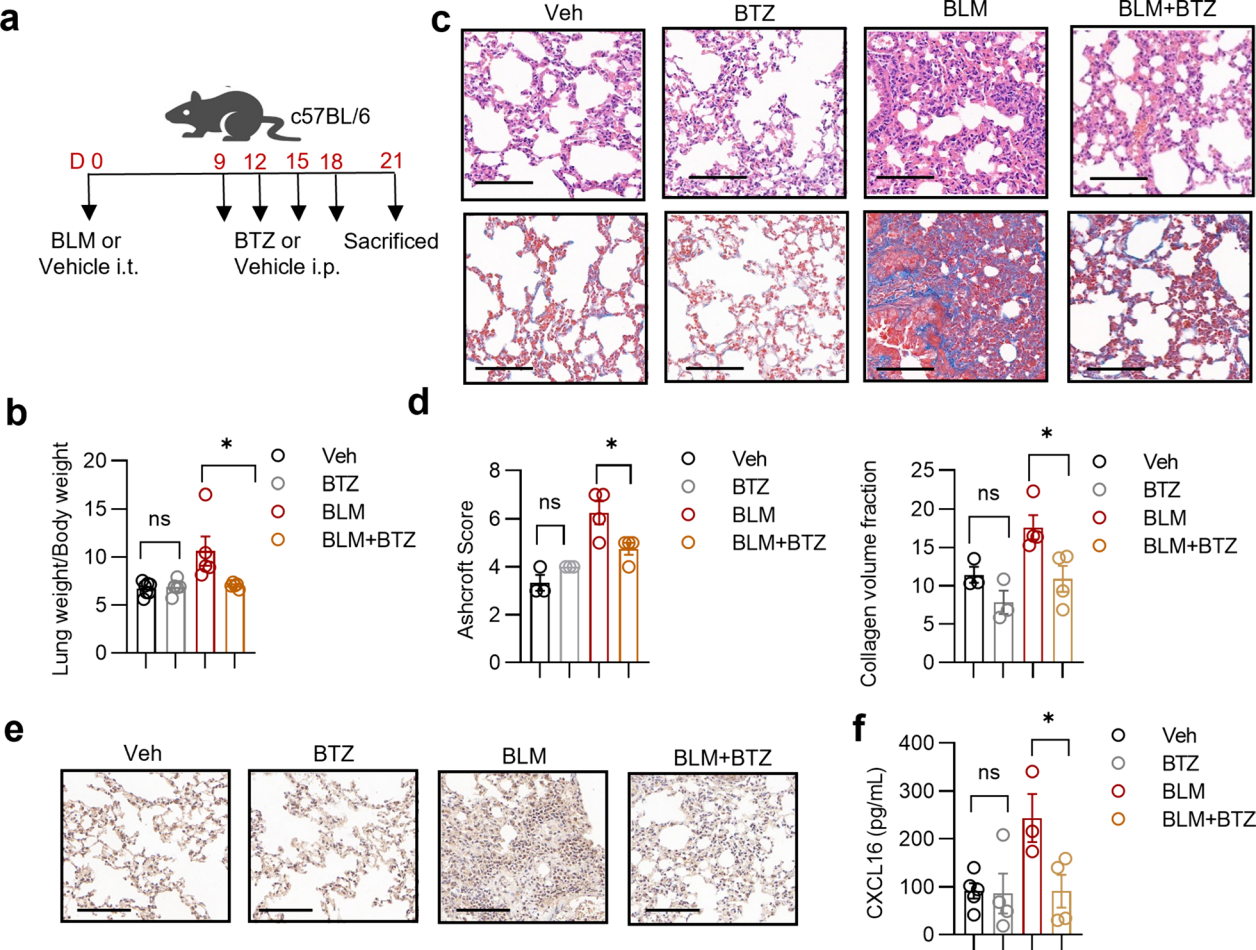
Bortezomib alleviates bleomycin-induced pulmonary fibrosis and suppresses CXCL16 expression. **a** The flow chart showing the process of the animal studies. **b** Summary bar graphs with scatter plots showing the lung weight (mg) /body weight (g) index evaluation of Veh group, BTZ group, BLM group, and BLM + BTZ group. **c** Representative sections of H&E staining and Masson’s Trichrome staining in indicated groups. Scale bar = 100 μm. **d** Summary bar graphs with scatter plots showing Ashcroft score evaluation of H&E staining and collagen volume fraction of Masson’s Trichrome staining in indicated groups. Scale bar = 100 μm. **e** Representative lung tissue sections of IHC staining of CXCL16. Scale bar = 100 μm. **f** Summary bar graphs with scatter plots showing CXCL16 secretion of PMA-stimulated pulmonary cell supernatant in indicated groups. *P < 0.05, ns, no significance. *Veh* vehicle, *BLM* bleomycin, *BTZ* bortezomib. Data are represented as mean ± SEM

Different from bleomycin, bortezomib, another chemotherapeutic drug acting as a proteasome inhibitor, exhibits protective potency in fibrotic diseases. We next investigated whether bortezomib alleviated lung fibrosis in vivo. Bortezomib was intraperitoneally dosed at 0.25 mg/kg every three days from day 9 to day 21 (Penke et al. 2022), as shown in Fig. 4a. As expected, intermittent administration of bortezomib significantly reduced lung weight/body weight index and relieved lung inflammation and collagen accumulation in bleomycin-induced pulmonary fibrosis mice, while bortezomib alone did not induced noticeable histological changes compared to vehicle (Fig. 4b–d). Next, we investigated whether bortezomib alleviated lung fibrosis through an immunoregulatory mechanism. Upon administration of bortezomib, CXCL16 expression of fibrotic lung tissues was markedly decreased, while lung tissues of the BTZ group did not show significant changes compared with those of the Veh group (Fig. 4e, f). Our data indicate the therapeutic effect of bortezomib in the mouse model of pulmonary fibrosis, which is associated with reduced CXCL16 secretion.

### Bortezomib suppresses M2 macrophages and CXCL16 secretion and diminishes CXCR6^+^ CD4 T chemotaxis in the context of BLM-induced pulmonary fibrosis

**Fig. 5:**
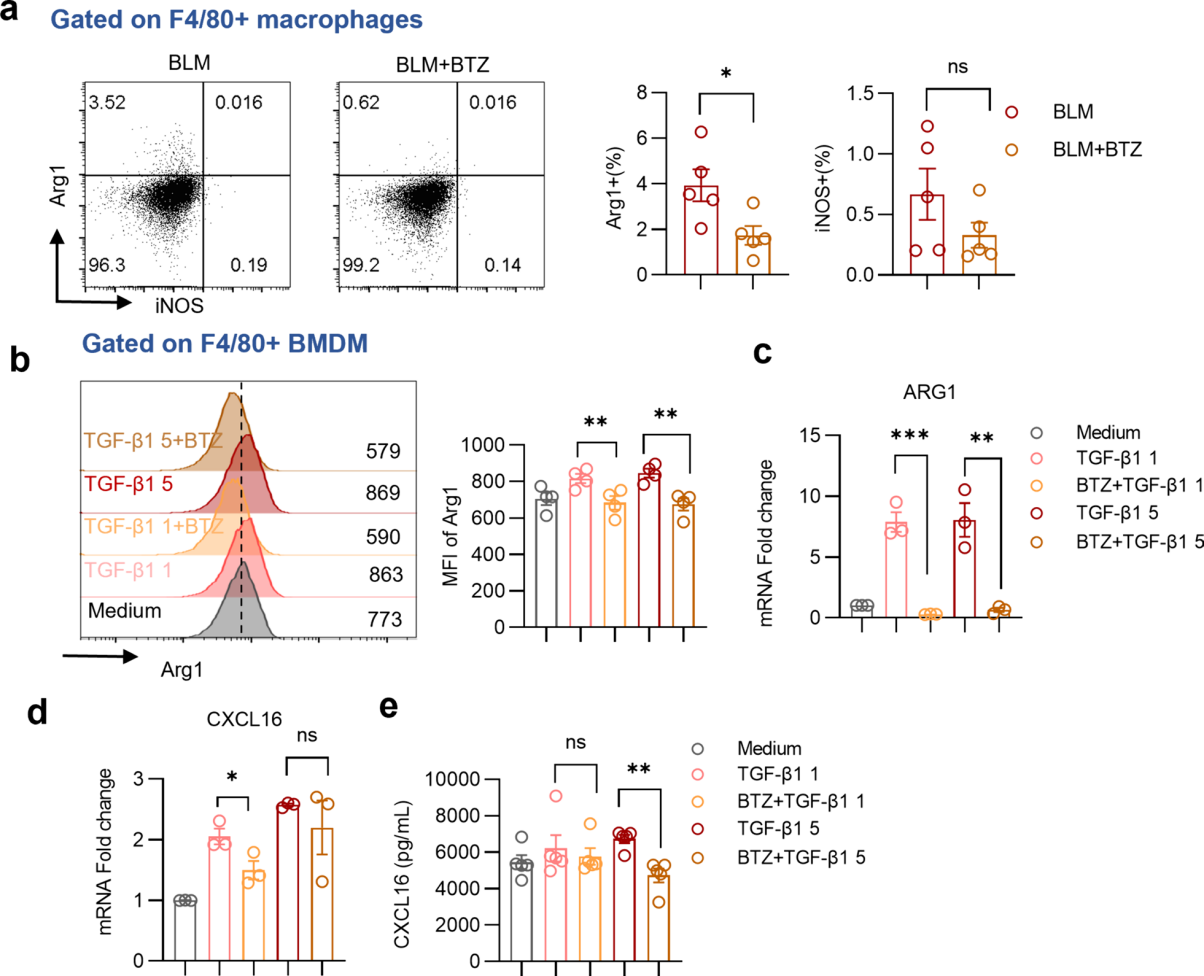
Bortezomib suppresses M2 macrophages and CXCL16 secretion. **a** Representative plots and summary bar graphs with scatter plots depicting the ratio of iNOS or Arg1 in pulmonary F4/80 + macrophages in BLM group and BLM + BTZ group. **b** Representative plots and summary bar graphs with scatter plots showing mean fluorescence intensity of Arg1 in F4/80^+^ BMDMs treated with 0, 1, 5 ng/ml recombinant murine TGF-β1 and 5 nM bortezomib. **c**, **d** Summary bar graphs with scatter plots showing ARG1 and CXCL16 mRNA expression in F4/80^+^ BMDMs treated with 0, 1, 5 ng/ml recombinant murine TGF-β1 and 5 nM bortezomib, respectively. **e** Summary bar graphs with scatter plots showing CXCL16 secretion of BMDM supernatant in indicated groups. *P < 0.05, **P < 0.01, ***P < 0.001, *ns* no significance. *BMDM* bone marrow-derived macrophages. *BLM* bleomycin, *BTZ* bortezomib. Data are represented as mean ± SEM

Given that bortezomib reduced CXCL16 secretion, we detected the impacts of bortezomib on M2-polarized macrophages. In the setting of in vivo fibrosis, bortezomib treatment markedly diminished Arg1 expression of pulmonary macrophages, as revealed by analysis of flow cytometry (Fig. 5a). In the context of TGF-β1 treatment, bortezomib reduced the protein level of Arg1 in BMDMs, as well as the mRNA expression of ARG1 (Fig. 5b, c). Simultaneously, in the culture medium supplemented with TGF-β1, cellular CXCL16 mRNA expression of BMDMs and CXCL16 concentration in the culture supernatant of BMDMs were restrained by bortezomib (Fig. 5d, e).

**Fig. 6:**
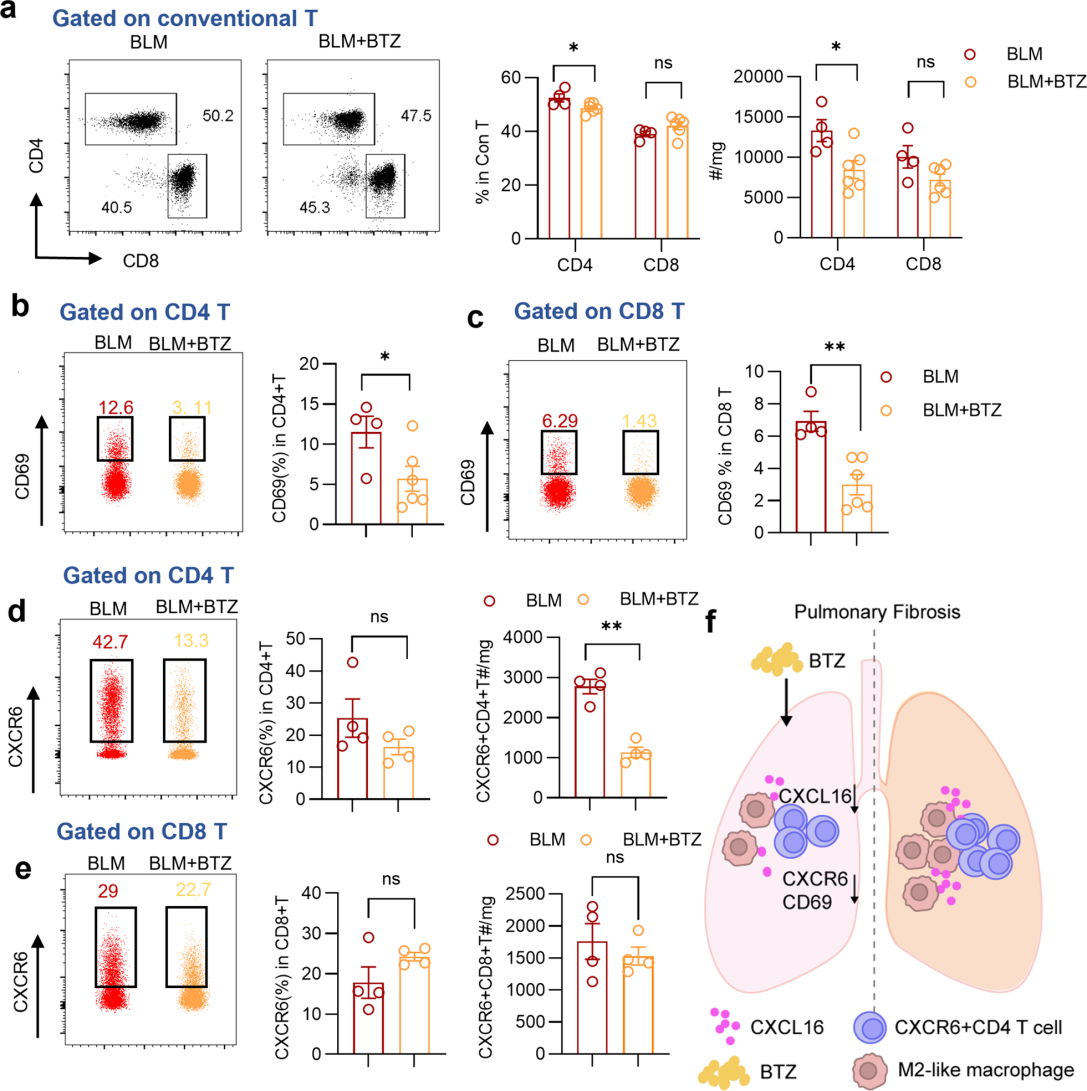
Bortezomib diminishes CXCR6^+^ CD4 T chemotaxis in the context of pulmonary fibrosis. **a** Representative plots and summary bar graphs with scatter plots depicting the ratio and number of CD4 T or CD8 T in pulmonary conventional T cells in BLM group and BLM + BTZ group. **b**, **c** Representative plots and summary bar graphs with scatter plots showing CD69 expression of CD4 T cells and CD8 T cells from indicated groups respectively. **d**, **e** Representative plots and summary bar graphs with scatter plots depicting the ratio and number of CXCR6^+^ CD4 T cells and CD8 T cells respectively. **f** Graph abstract of the role of BTZ in pulmonary fibrosis. *P < 0.05, **P < 0.01, *ns* no significance. *BLM* bleomycin, *BTZ* bortezomib. Data are represented as mean ± SEM

On the other hand, accumulation of CD4 T cells was observed to be eliminated, as evidenced by the decreased population and number of CD4 T cells in the BLM + BTZ group versus the BLM group, while no significant changes were observed in CD8 T cells (Fig. 6a). Additionally, after treatment with bortezomib, CD69 expression and CXCR6 expression were coincidently downregulated in CD4 T cells (Fig. 6b, d). Meanwhile, in CD8 T cells, only activation levels exhibited a decrease (Fig. 6c, e). These findings collaboratively reveal that bortezomib diminishes pulmonary fibrosis by immunoregulatory mechanism, in which suppression of M2 polarization may mediate downregulation of CXCL16 production, thus contributing to impaired accumulation of CXCR6^+^ CD4 T cells (Fig. 6f).

## Discussion

Emerging researches have emphasized the role of chemokines in pulmonary fibrosis, including CC Chemokines (Liu et al. 2023, 2021) and CXC family (Strieter et al. 2007). Chemokines, such as CXCL12 (Antoniou et al. 2010; Chow et al. 2016), CCL1 (Liu et al. 2021), and CCL18 (Prasse et al. 2006), are implicated in pulmonary fibrosis, acting as chemoattractants by combining with the chemokine receptors, and yet the role of CXCL16 and its receptor remain poorly understood. A recent study has demonstrated that upregulation of serum CXCL16 levels in patients with rheumatoid arthritis-associated pulmonary fibrosis stimulates proliferation, migration, and collagen production of fibroblasts via the PI3K/AKT/FOXO3a pathway (Ma et al. 2020). Besides, CXCL16 is implicated in promoting epithelial-mesenchymal transition in alveolar type II-like epithelial cells by TGF-β1/Smad3 signaling (Ma et al. 2021). In our current study, we observe an upregulation of CXCL16 in the fibrotic lung tissues of mice and investigate its cellular source. We find that M2-polarized macrophages show highest mRNA expression of CXCL16 in pulmonary cell subsets, potentially contributing to the recruitment of CXCR6^+^ CD4 T cells. However, it's worth noting that we do not analyze the protein level of CXCL16 in all subsets or specifically discuss the expression of membrane-bound and secretory forms of CXCL16 in this study.

The diverse phenotypes of macrophages exert varying effects in tissue injury, repair, and fibrosis partly due to their unique cytokine and chemokine profiles. Pro-inflammatory macrophages (M1 phenotype) are responsible for early-stage lung inflammation and injury, whereas alternatively activated macrophages (M2 phenotype) are associated with fibrotic manifestations (Wynn and Vannella 2016). A study have shown that depletion of macrophages suppresses the progress of pulmonary fibrosis, which is related to reduction of Arg1 expression, highlighting the detrimental effects of the M2 macrophages (Gibbons et al. 2011). The profibrogenic effect of M2 macrophages involves the regulation of epithelial-mesenchymal transition (EMT) and fibroblast-myofibroblast transition (FMT). M2 macrophages produce TGF-β1 to activate the TGF-β/Smad2 signaling pathway, thereby promoting EMT progress (Zhu et al. 2017). Moreover, myofibroblast differention can also be induced by M2 macrophages, which is associated with Wnt/beta-catenin signaling (Hou et al. 2018). In addition, M2 macrophages produce multiple mediators, such as chemokines and cytokines, to amplify fibrosis progress by mediating interaction with other immune cells. For instance, CCL22 and CCL17, secretd by M2 macrophages, can recruit CCR4^+^CD4^+^ T helper 2 cells, thereby contributing to the pathophysiology of pulmonary fibrosis (Inoue et al. 2004; Kishore and Petrek 2021). Our study further highlights the accumulation of Arg1^+^ M2 macrophages in both in vivo and in vitro fibrotic contexts, and underscores the pivotal role of CXCL16-secreting Arg1^+^ M2 macrophages in the recruitment of CXCR6^+^ CD4 T cells..

The retention and recruitment of CD4 T cells play a pivotal role in the exacerbation or remission of pulmonary fibrosis. Bronchoalveolar lavage samples from patients with pulmonary fibrosis have shown an accumulation of CD4 T cells producing IFN-γ and IL-13 (Sikkeland et al. 2021), consist with that, another study demonstrated that CD103^low^ CD4^+^ tissue-resident memory cells that produced IFN-γ, IL-5, and IL-13 promoted fibrotic responses in mice chronically exposed to *Aspergillus fumigatus* (Ichikawa et al. 2019). Conversely, CD69^hi^ CD103^hi^ Foxp3^+^ CD4^+^ regulatory T cells restrained pathogenic populations. Accordingly, adoptive transfer of CCR2^+^CD4 T cells, which contain a high ratio of Foxp3^+^CD25^+^ T cells, attenuate lung inflammation and fibrosis (Milger et al. 2017). In the study, we demonstrate conventional CD4 T cells but not CD8 cells from fibrotic lung tissues display higher expression of CD69 and CXCR6 simultaneously, which is associated with CXCL16-mediated recruitment. However, the precise function of CXCR6^+^ CD4 T cells remains unclear and warrants further detailed investigation.

Bortezomib, approved as an antineoplastic agent, inhibits the proteasome, disrupts cellular homeostasis, and induces cell death (Richardson et al. 2006). It targets the ubiquitin–proteasome pathway by inhibiting the 20S proteasome core proteolytic activities (Gazzaroli et al. 2023). However, previous evidence suggests that therapeutic administration of bortezomib inhibits fibroblast activation and promotes myofibroblasts dedifferentiation and apoptosis without reducing proteasome activity (Penke et al. 2022). Emerging evidence supports anti-fibrosis potency of bortezomib, extending beyond its effects on fibroblasts. Depletion of plasma cells by bortezomib, but not anti-CD20 B-cell ablation, mitegates the fibrosis progress induced by bleomycin (Prêle et al. 2022). In the study, we demonstrate a similar anti-fibrosis effect of bortezomib in bleomycin-induced pulmonary fibrosis model and reveal an immunoregulatory mechanism that bortezomib significantly decreases the ratio and quantity of Arg1^+^ macrophages and restrains subsequent CXCL16 secretion-associated CXCR6^+^ CD4 T accumulation, suggesting that M2 macrophages may show high sensitivity to bortezomib. Additionally, a previous study has indicated increased ARG1 expression in peripheral blood and bone marrow of bortezomib-refractory multiple myeloma patients,observed that in bortezomib-refractory multiple myeloma patients, ARG1 was increased in peripheral blood and bone marrow, suggesting that arginase-induced arginine deprivation may protect the myeloma cell from bortezomib-induced apoptosis (Giallongo et al. 2022). However, the exact reasons for the effects of bortezomib on M2 macrophages remain unknown. Nevertheless, our study provides preclinical evidence for the drug repurposing strategy of bortezomib in pulmonary fibrosis.

## Conclusions

In summary, our research revealed the increased secreation of CXCL16 from M2 macrophages in the context of bleomycin-induced pulmonary fibrosis, which was associated with the recruitment of CXCR6^+^ CD4 T cells. In addition, bortezomib exhibited anti-fibrosis effects by decreasing Arg1^+^ macrophages and restrained subsequent CXCL16-associated CXCR6^+^ CD4 T accumulation. Our findings provide insights into the pathogenesis of pulmonary fibrosis and suggest that bortezomib may be a promising treatment for pulmonary fibrosis, especially in cases accompanied by malignancy.

### Supplementary Information

Supplementary Material 1: Figure S1: **a** Heatmap showing correlation between core enriched genes expression of chemokine signaling pathway and TGFB1 gene expression. **b** Plots showing Gating strategy of F4/80 + macrophages. **c** Plots showing Gating strategy of conventional T cells.

## Data Availability

All data generated or analysed during this study are included in this article and the GSE datasets analysed during the current study are available in the GEO Database (https://www.ncbi.nlm.nih.gov/gds/).

## Abbreviations

Arg1: Arginase 1
BLM: Bleomycin
BTZ: Bortezomib
BMDM: Bone marrow-derived macrophages
ELISA: Enzyme-Linked Immunosorbent Assay
EMT: Epithelial-mesenchymal transition
FBS: Fetal bovine serum
FMT: Fibroblast-myofibroblast transition
GSEA: Gene Set Enrichment Analysis
HE: Haematoxylin–eosin
IL: Interleukin
iNKT: Invariant natural killer T cell
iNOS: Inducible nitric oxide synthase
IHC: Immunohistochemistry
Ion: Ionomycin
MFI: Mean fluorescence intensity
PMA: Phorbol myristate acetate
PBS: Phosphate-buffered saline
RT-qPCR: Quantitative reverse transcription polymerase chain reaction
Veh: Vehicle
