# Supplementary figures and images for: Bortezomib restrains M2 polarization and reduces CXCL16-associated CXCR6+CD4 T cell chemotaxis in bleomycin-induced pulmonary fibrosis
Source: Mol Med. 2024 May 24;30:70. doi: 10.1186/s10020-024-00836-5 (PMC11127379; doi:10.1186/s10020-024-00836-5)

# Supplementary Figure. 1

**a** GSE213709

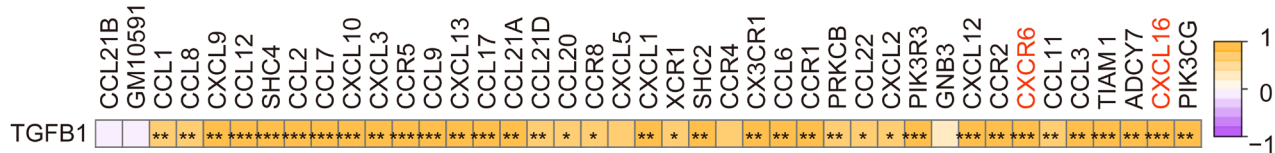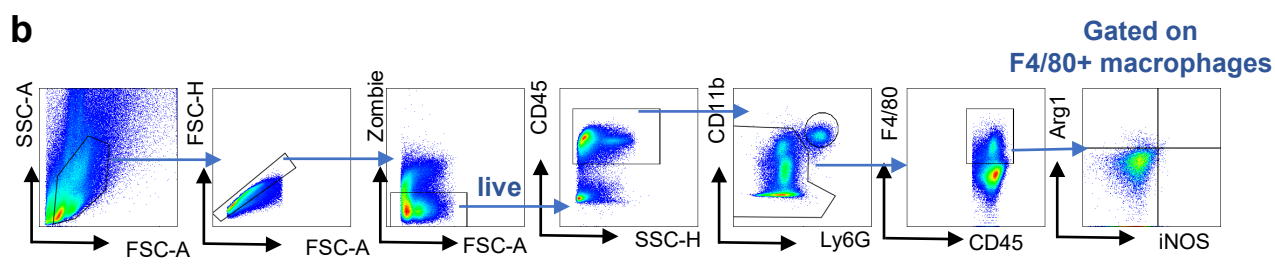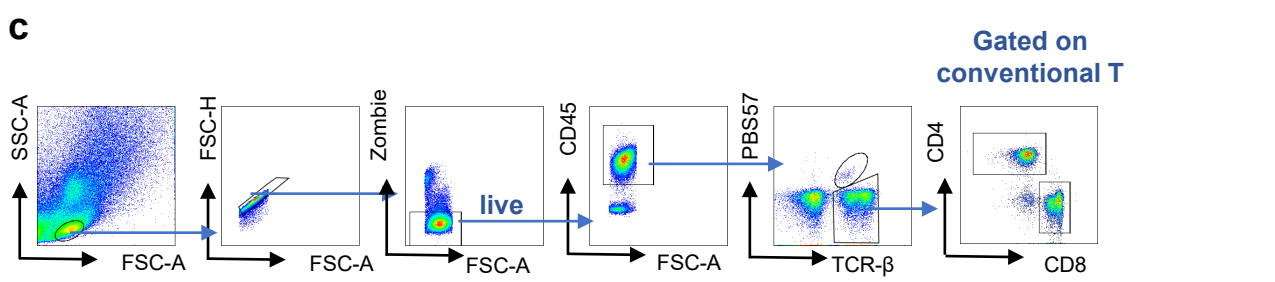

Supplement: Supplementary file 1 — Supplementary Material 1: Figure S1: a Heatmap showing correlation between core enriched genes expression of chemokine signaling pathway and TGFB1 gene expression. b Plots showing Gating strategy of F4/80 + macrophages. c Plots showing Gating strategy of conventional T cells. [file 10020_2024_836_MOESM1_ESM.pdf]
